# Isolated ileocolic artery occlusion presented with segmental bowel infarction: a case report

**DOI:** 10.1186/1757-1626-2-9153

**Published:** 2009-12-07

**Authors:** Hany M El Hennawy, Mohamed Fahmy Abdalla, Abdelrahman El-Osta, Elsaid MA Bedair

**Affiliations:** 1 Department General Surgery, Al Khor Hospital, Hamad Medical Corporation, Doha, PO Box 3050, Qatar; 2 Department of Radiology, Al Khor Hospital, Hamad Medical Corporation, Doha, PO Box 3050, Qatar

## Abstract

Acute mesenteric ischemia is a serious acute abdominal condition requiring early diagnosis and intervention to improve the outcome. Although transmural acute bowel infarction represents about 1% of all cases of acute abdomen, it has a higher annual mortality rate than colon cancer. It tends to affect the colon in segmental fashion, mostly the splenic flexure and rectosigmoid portions of the colon. Isolated ischemia of the right side of the colon is rarely reported, especially in association with shock. Diagnosis of acute colonics ischemia is challenging as it may easily be confused with other non ischemic conditions both clinically and radiologically. Surgical resection is still the main curative approach. We present a case of segmental terminal ileum, cecum and part of ascending colon infarction due to isolated IleoColic artery thrombosis.

## Case presentation

**Figure 1:**
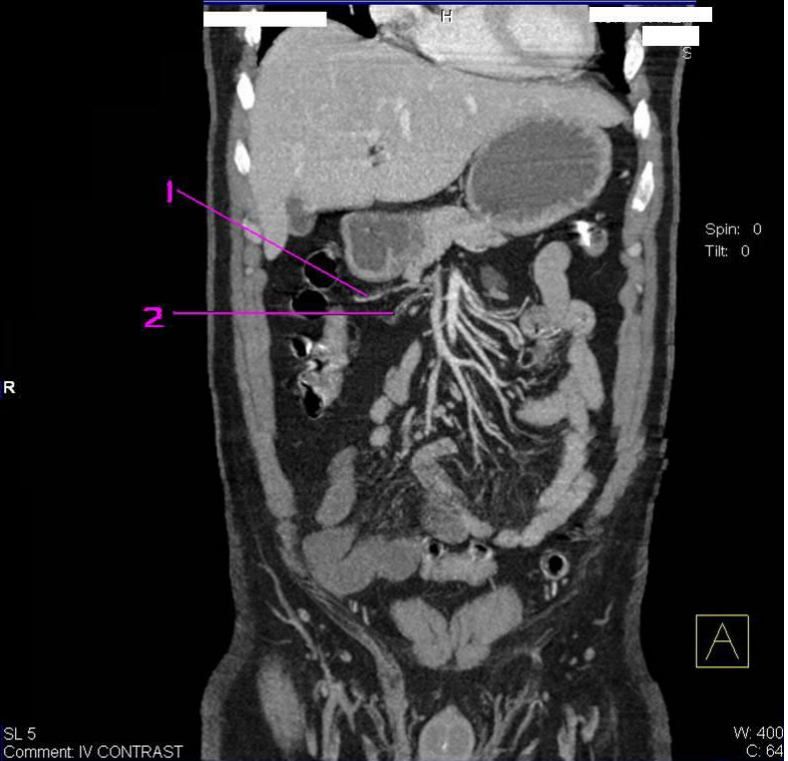
**Abdominal Angio CT coronal reconstructed image revealed; no enhancement of the distal IleoColic artery in arterial phase denoting complete thrombosis**. Note also; decreased degree of venous enhancement in the IleoColic vein as an effect of arterial thrombosis. (1) IleoColic vein. (2) IleoColic artery.

**Figure 2:**
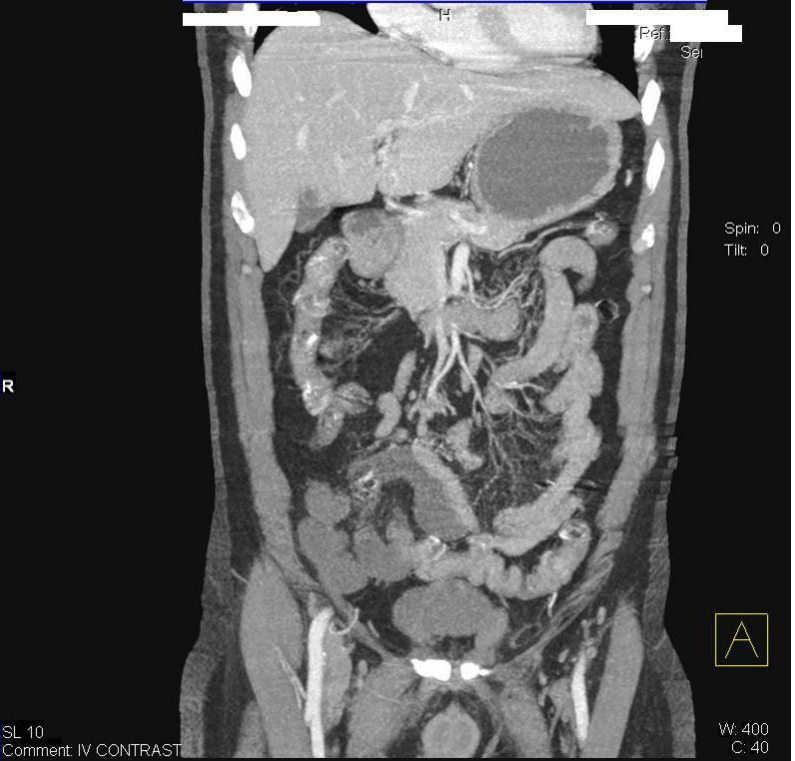
**Abdominal Angio CT coronal reconstructed image revealed; marked discrepancy of the bowel wall enhancement between the distal ileum and right colon, and the proximal ileal loops and left colon, denoting arterial ischemia**.

A 57-year-old Indian male laborer was admitted to the hospital from the Emergency Department complaining of central abdominal pain. The pain had started on the previous day with severe colicky pain starting around the umbilicus. This pain was non-radiating, was not related to meals. It was associated with anorexia, nausea, one attack of vomiting [coffee ground vomitus], and two attacks of non bloody diarrhea with no mucus. There was no history of urinary symptoms, fever or weight loss. The patient was hypertensive on beta blockers, had no history of surgeries or allergy, and had no special habits. Physical examination revealed no evidence of arrhythmia or heart failure. Physical examination was significant only for marked generalized abdominal distension, tenderness, rebound tenderness, abdominal guarding and rigidity. There were no bowel sounds, no ascites or organomegaly. Rectal examination revealed an empty rectum with mild dark blood in the rectum. Blood workups showed some abnormal limits; white blood cell count of 13.5 thousand/ml, sodium 129 mmol/L, potassium 5 mmol/L and bilirubin 50 umol/L. Urine examinations was positive for ketones, glucose and red blood cells. Chest x-ray revealed no air under the diaphragm or signs of intestinal obstruction. Angio CT of the Abdomen revealed complete thrombosis of the distal IleoColic artery with secondary nonenhancement of the wall of the distal ileum, cecum and part of ascending colon secondary to arterial occlusion (thrombosis). [Figure 1, and Figure 2]. A bolus of intravenous heparin sodium was given to avoid any further thrombus propagation. The patient consented and was prepared for an urgent exploratory laparotomy which revealed gangrene affecting 5 cm of the distal ileum and cecum and about 6 cm of the ascending colon. Careful examination found both small and large intestine intact. A right hemicolectomy was performed with Ileo-transverse anastomosis. Histopathology examination revealed black discoloration of the distal 5 cm of terminal ileum, Cecum and the proximal 6 cm of the ascending colon with no visible perforations. Microscopic examination revealed submucocal and transmural necrosis of the same segments of terminal ileum, cecum and proximal ascending colon. Appendix showed small organizing thrombus in the submucosal vessels and both resection margins were viable. Post operative Lab work up revealed prolonged prothrombin time (13.2 seconds), increased lactic acid level (3.6 mmol/L), increased D Dimer automated (417 ng/ml), positive C-Reactive protein (96 mg/L), Antithrombin lll function deficiency (61%), normal protein C clotting and protein S clotting, normal activated protein C resistance test, normal carcinoembryonic antigen (CEA), CA 19-9, homocysteine, and prostate specific antigen (PSA) normal fibrinogen level, negative anticardiolipin Ig M, ANA, ANCA ethanol. Abdominal Angio CT was done on the second post operative day and was normal. Patient was discharged home after 8 days.

## Discussion

Acute mesenteric ischemia represents one of the most threatening abdominal conditions in elderly patients [1]. It has high mortality rate (50%-90%, depending on the cause of the event and the degree and extent of ischemic bowel wall damage despite medical advances [2]. Most cases of acute intestinal ischemia result either from thrombosis of a preexisting stenotic lesion or from embolization [3] (most frequently to the SMA). Cardiac emboli are the most common variety, though tumor emboli [4], and atheroemboli are seen as well. Atheroemboli generally result from iatrogenically induced cholesterol embolization caused by aortic catheterization. Acute mesenteric artery thrombosis accounts for 25% to 30% of all ischemic events[4]. Segmental ischemia of the right side of the colon is uncommon and reported particularly in case of shock [5]. Acute occlusions of the superior mesenteric artery due to thrombosis or embolization are responsible for approximately 60%-70% of cases of acute bowel ischemia, Acute occlusions of the mesenteric arteries may be related to numerous other conditions, however, including atherosclerosis, thromboembolism from the aorta, mesenteric arterial thrombosis, aortic or mesenteric arterial dissection, spontaneous or postoperative cholesterol embolization, aortic surgery, stent placement, or therapeutic embolization of mesenteric vessels to treat gastrointestinal hemorrhage [6]. Isolated infarction of cecum was reported in two patients who were both diabetic and hypertensive [5]. In contrast to the dual blood supply serving the ileum, appendix and ascending colon, the cecum is supplied by end arteries which may render it more susceptible to ischemia [5]. Also poor collateral vessel flow may put the cecum at risk for embolic infarction commonly by atheromatous emboli [7]. In subject patient there is associated Antithrombin lll function deficiency (61%), hypofibrinogemia and increased D Dimer which indicate hypercoagulable state. CT scan is a fast, widely available noninvasive modality that holds great promise for use in the diagnosis of AMI. CT

Diagnostic criteria of AMI include pneumatosis intestinalis, venous gas, superior mesenteric artery occlusion, celiac and inferior mesenteric artery arterial occlusion with distal superior mesenteric artery disease, or arterial embolism or, alternatively, bowel wall thickening combined with any one finding of focal lack of bowel wall enhancement, solid organ infarction, or venous thrombosis. By combining these criteria, a sensitivity of 96% and a specificity of 94% can be achieved [8]. Other CT findings suggestive of bowel ischemia include thumbprinting, intramral hemorrhage, focal or diffuse bowel dilatation, mesenteric arterial thrombus, engorged mesentery, portal or mesenteric venous gas and pneumoperitoneum [9]. Unfortunately, common CT findings in bowel ischemia are not specific, and specific findings are rather uncommon [10]. CT findings of isolated cecal infarction may be mistaken for typhlitis; ischemia of the terminal ileum or segmental bowel ischemia, Crohn disease; ischemic pancolitis and infectious or ulcerative colitis. However, the most common cause of CT misinterpretation of non ischemic conditions for bowel ischemia is the presence of pneumatosis or portal venous gas [11]. Contrast-enhanced ultrasonography is a promising tool for the assessment of bowel ischemia in patients with radiographic evidence of small-bowel dilatation. By taking the noninvasive nature of the test into account, this method has potential for application in daily practice as a diagnostic method for bowel ischemia [12]. Operative intervention remains the mainstay of management of almost all patients who present with AMI. Surgery is indicated in all patients with peritonitis after rapid resuscitation. At laparotomy the surgeon should establish the diagnosis, consider appropriate revascularisation and resect the already damaged bowel. The decision for relook laparotomy should be made at the initial surgery itself and is independent of the clinical status of the patient between the two procedures [13]. The goals in the surgical treatment of acute mesenteric ischemia are (i) to restore normal pulsatile flow to the SMA and (ii) to resect any nonviable intestine. In general, revascularization precedes resection. The therapeutic approach varies, depending on the specific underlying cause. For embolic disease of the SMA, the standard treatment is surgical embolectomy using the balloon catheter thromboembolectomy, with or without patch angioplasty of the superior mesenteric artery [14]. Management of Superior Mesenteric Artery Thrombosis is performed by thrombectomy, if possible, or (usually) by a bypass. Options include an aortovisceral graft (with a polyester fluoroethylene prosthesis or the saphenus vein) either antegrade from the supraceliac or retrograde from the infraceliac aorta or iliomesenteric bypass grafting originating from the right or left iliac artery [15]. In most cases, a single-vessel, retrograde bypass graft is best since it requires the least time to complete and causes less hemodynamic disturbance through cross-clamping of the iliac artery [16]. Percutaneous Transcatheter Thromboaspiration is another treatment modality of Superior Mesenteric Artery Thrombosis;An 8F guiding catheter (Cordis, Roden, the Netherlands) placed into the ostium of superior mesenteric artery from a percutaneous transfemoral approach, and embolectomy performed coaxially with 7F and 6F aspiration catheters using a 60-mL locking syringe for aspiration [17]. After successful restoration of mesenteric arterial flow, laparoscopic examination of the viability of the abdominal organs was performed. Intensive anticoagulation was maintained. Thrombolysis of the SMA thrombosis through intra-arterial infusion of urokinase or streptokinase may result in revascularization of the small intestine. Angiographic demonstration of the arterial blood flow with venous return in the small mesenteric vessels also is the superior monitorization method for the intestinal viability. Therefore, patency should be checked 30 minutes after intra-arterial infusion of thrombolytic agent with an opaque injection associated with power Doppler ultrasound scan. If repeated angiography shows occlusion, laparotomy should be performed urgently. Surgical resection of necrotic bowel and anticoagulant therapy to control progressive vascular thrombosis should be carried out without delay. Time-consuming radiological and laboratory investigations should be avoided [18].

Some investigators have promoted endovascular treatment as a first-line therapy for mesenteric occlusive disease [19]. Simonetti and associates have reported on seven patients who presented with acute occlusive mesenteric ischemia [20]. However, only five cases were amenable to angioplasty, fibrinolysis, or both. Of those five patients, four showed clinical improvement without need for operation. Other groups have reported even more limited success in the endovascular management of patients with acute mesenteric ischemia [21,22]. Endovascular treatment of patients with acute mesenteric ischemia also may expose the patient to the risk of ongoing ischemic damage during the wait for the thrombolytic therapy to have an effect. Furthermore, acute bowel ischemia may result in mucosal slough, leaving a large raw surface area, so that infusion of thrombolytic agents directly into the superior mesenteric artery vessel could result in significant gastrointestinal hemorrhage. Finally, the status of the bowel integrity cannot be addressed through angiographic techniques, and early recognition and resection of ischemic bowel that may progress to perforation are essential to avoid potentially disastrous clinical deterioration [23]. Percutaneous intervention followed by laparoscopic evaluation of bowel integrity as a minimally invasive approach to mesenteric ischemia has been reported. Currently, the best candidates for endovascular treatment of acute mesenteric ischemia may be patients with angiographic findings of good collateral circulation, minimizing the chance of bowel infarction during attempted thrombolysis. Thus, at present, despite the appeal of treating these very ill patients in a "less invasive" manner, support for the safety and efficacy of such an approach is lacking [24].

Laparoscopic second-look operation is one of the safest interventions, which proves the technique is really "minimally invasive." It not only prevents unnecessary laparotomies but also saves poor-risk patients from additional surgical trauma. It has become the routine procedure of choice for every patient who is operated on with the diagnosis of mesenteric ischemia [25].

## Conclusion

Acute arterial mesenteric ischemia is a serious condition if not recognized and treated early. There is often a delay in diagnosis and treatment of this condition because of its diverse causes and non specific radiological studies. We recommend that whenever mesenteric ischemia is suspected Angio CT should be ordered because the routine CT examinations may miss focal arterial mesenteric ischemia. Decreasing the time to diagnosis remains the only reliable means to decrease the morbidity and mortality associated with this disease.

## Abbreviations

AMI: acute mesenteric ischemia; ANA: antinuclear antibody; ANCA: anti-neutrophilic cytoplasmic antibodies; CA 19-9: cancer antigen 19-9; CEA: carcinoembryonic antigen; CT: computerized tomography; PSA: prostate specific antigen; SIRS: systemic inflammatory response syndrome.

## Consent

Written informed consent was obtained from the patient for publication of this case report and accompanying images. A copy of the written consent is available for review from the journal's Editor-in-Chief.

## Competing interests

The authors declare that they have no competing interests.

## Authors' contributions

HH, MFA, AEO did the surgery. HH, EMB wrote the article. All authors read and approved the final version of the manuscript.

## References

[B1] Jrven O Laurika J Salenius JP Tarkka M Acute intestinal ischemia: a review of 214 cases Ann Chir Gynaecol 1994 83 22 25 8053632

[B2] Brandt L Boley S Goldberg L Mitsudo S Bergman A Colitis in the elderly Am J Gastroenterol 1981 76 239 245 7315820

[B3] Taylor LM Moneta GL Porter JM Treatment of acute intestinal ischemia caused by arterial occlusions Rutherford Vascular Surgery 2000 5 Philadelphia: WB Saunders Co 1512 1518

[B4] Low DE Frenkel VJ Manley PN Ford SN Kerr JW Embolic mesenteric infarction: a unique initial manifestation of renal cell carcinoma Surgery 1989 106 925 928 2683176

[B5] Bower TC Ischemic colitis Surg clin North Am 1993 73 1037 1053 8378827 10.1016/s0039-6109(16)46139-6

[B6] Oldenburg WA Lau LL Rodenberg TJ Edmonds HJ Burger CD Acute Mesenteric Ischemia: A Clinical Review Arch Intern Med 2004 164 10 1054 1062 10.1001/archinte.164.10.1054 15159262

[B7] Simon AM Birnbaum BA Jacobs JE Isolated infarction of the cecum: CT findings in two patients Radiology 2000 214 513 516 10671602 10.1148/radiology.214.2.r00fe15513

[B8] Ando M Ito M Mishima Y Spontaneous dissecting aneurysm of the main trunk of the superior mesenteric artery:report of a case Surg Today 1995 25 468 470 10.1007/BF00311831 7640482

[B9] Chan T Levine MS Park Y cholesterol embolization as a cause of cecal infarct mimicking carcinoma AJR Amz J Roentgenol 1988 150 6 1315 1316 10.2214/ajr.150.6.1315 3259371

[B10] Kirkpatrick ID Kroeker MA Greenberg HM Biphasic CT with Mesenteric CT Angiography in the Evaluation of Acute Mesenteric Ischemia:Initial Experience Radiology 2003 229 91 98 10.1148/radiol.2291020991 12944600

[B11] Alpern MB Glazer GM Francis IR Ischemic or infracted bowel: CT findings Radiology 1988 166 149 152 3336673 10.1148/radiology.166.1.3336673

[B12] Hata J Kamada T Haruma K Kusunoki H Evaluation of Bowel Ischemia with Contrast-enhanced US: Initial Experience Radiology 2005 236 2 712 715 10.1148/radiol.2362040299 16040926

[B13] Park W Contemporary management of acute mesenteric ischemia: Factors associated with survival J Vasc Surg 2002 35 3 445 452 10.1067/mva.2002.120373 11877691

[B14] Cikrit DF Harris VJ Hemmer CG Kopecky KK Dalsing MC Hyre CE Fischer JM Lalka SG Sawchuk AP Comparison of spiral CT scan and arteriography for evaluation of renal and visceral arteries Ann Vasc Surg Mar 1996 10 2 109 116 10.1007/BF02000753 8733861

[B15] Taylor LM Jr Porter JM From Treatment of acute intestinal ischemia caused by arterial occlusions Rutherford Vascular Surgery 1995 4 Philadelphia: Saunders Co 1274 1284

[B16] Michael U Melina R Chicago I Mesenteric Ischemia Perspect Vasc Surg Endovasc Ther 2005 17 317 318 10.1177/153100350501700405 16389425

[B17] Hladik P Treatment of acute mesenteric thrombosis/ischemia by transcatheter thromboaspiration J Surg 2005 137 122 123 10.1016/j.surg.2004.05.053 15614294

[B18] Oguzkurt P Mesenteric Vascular Occlusion Resulting in Intestinal Necrosis in Children J Pediatr Surg 2000 35 1161 1164 10.1053/jpsu.2000.8718 10945686

[B19] Steinmetz E Tatou E Favier-Blavoux C Bouchot O Cognet F Cercueil JP Krause D David M Brenot R Endovascular treatment as first choice in chronic intestinal ischemia Ann Vasc Surg 2002 16 693 699 10.1007/s10016-001-0321-3 12391508

[B20] Simonetti G Lupattelli L Urigo F Barzi F Mosca S Maspes F Guazzaroni M Interventional radiology in the treatment of acute and chronic mesenteric ischemia Radiol Med (Torino) 1992 84 98 105 1387237

[B21] Calin GA Calin S Ionescu R Croitoru M Diculescu M Oproiu A Successful local fibrinolytic treatment and balloon angioplasty in superior mesenteric arterial embolism: A case report and literature review Hepatogastroenterology 2003 50 732 734 12828073

[B22] Yamaguchi T Saeki M Iwasaki Y Ishikawa M Hayakawa M Sakuyama K Ishikawa T Ashida H Local thrombolytics therapy for superior mesenteric artery embolism: Complications and long-term clinical follow-up Radiat Med 1999 17 27 33 10378649

[B23] Keller TT Rutherford Vascular Surgery 2005 6 Philadelphia: Elsevier 1265 1269

[B24] Leduc FJ Pestieau SR Detry O Hamoir E Honoré P Trotteur G Jacquet N Acute mesenteric ischaemia: Minimal invasive management by combined laparoscopy and percutaneous transluminal angioplasty Eur J Surg 2000 166 345 347 10.1080/110241500750009221 10817335

[B25] Anadol AZ Ersoy E Taneri F Tekin EH Laparoscopic "Second-Look" in the Management of Mesenteric Ischemia Surg Laparosc Endosc Percutan Tech 2004 4 191 193 10.1097/01.sle.0000136677.39377.62 15472545

